# Chemoradiotherapy as Definitive Treatment for Elderly Patients with Head and Neck Cancer

**DOI:** 10.1155/2018/3508795

**Published:** 2018-01-17

**Authors:** Jens Müller von der Grün, Daniel Martin, Timo Stöver, Shahram Ghanaati, Claus Rödel, Panagiotis Balermpas

**Affiliations:** ^1^Department of Radiotherapy and Oncology, J. W. Goethe University, Frankfurt am Main, Germany; ^2^Department of Otorhinolaryngology, J. W. Goethe University, Frankfurt am Main, Germany; ^3^Department of Maxillofacial Surgery, J. W. Goethe University, Frankfurt am Main, Germany; ^4^German Cancer Research Center (DKFZ), Heidelberg, Germany; ^5^German Cancer Consortium (DKTK), Partner Site Frankfurt, Frankfurt, Germany

## Abstract

**Background:**

With the aging population and a rising incidence of squamous cell carcinoma of the head and neck (SCCHN), there is an emerging need for developing strategies to treat elderly patients.

**Patients and Methods:**

We retrospectively analyzed 158 patients treated with definitive, concurrent chemoradiotherapy (CRT) for SCCHN. Clinicopathological characteristics, acute toxicities, and oncological outcomes were compared between patients younger and older than (or of age equal to) 65, 70, and 75 years.

**Results:**

RT dose, chemotherapy regimen, and total chemotherapy dose were balanced between the groups. After a median follow-up of 29 months, overall survival (OS), progression-free survival (PFS), local control rate, and distant metastasis-free survival stratified by age of ≥65, ≥70, or ≥75 years revealed no differences. The rate of acute toxicities was also not higher for older patients. Worse ECOG performance score (ECOG 2-3) was associated with impaired OS (*p* = 0.004) and PFS (*p* = 0.048).

**Conclusion:**

Definitive treatment with CRT for SCCHN is feasible and effective; even in advanced age treatment decisions should be made according to general condition and comorbidity, rather than calendar age alone.

## 1. Introduction

Squamous cell carcinoma of the head and neck (SCCHN) is diagnosed about 500.000 times each year worldwide with rising incidence [1]. This growing number is related to younger individuals with Human-Papilloma-Virus- (HPV-) positive tumors and to an increasing number of elderly patients due to a generally increasing life expectancy in Western societies [2]. SCCHN usually peaks within the sixth decade of life and is accompanied by tobacco and/or alcohol misuse [3]. Treatment of SCCHN is, depending on curative or palliative intention and organ preservation or not, single- or combined-modality treatment including surgery, radiotherapy (RT), and chemotherapy. The standard of organ preserving treatment for locally advanced SCCHN is chemoradiotherapy (CRT) with 70 Gy and concurrent platin-based chemotherapy [4]. In total, these therapies can take up to 8–12 weeks for surgery plus adjuvant RT/CRT or 7 weeks for definitive CRT. The therapeutic management in elderly patients is challenging as their possible comorbidities, poor performance, and/or mental status might hamper the realization of aggressive, curative treatment approaches [5]. Although platinum-based CRT has shown advances in terms of locoregional control (LRC) and overall survival (OS) in randomized controlled trials and meta-analyses, these benefits are often accompanied by worse acute and late-term toxicities [6, 7], which makes the implementation of such combined-modality therapies for elderly and frail patients challenging.

With rising life expectancy, also the number of elderly patients with good performance status and without impairing factors increases. Therefore, although chronological age remains an important factor, the emphasis on patients' biological age for decision making in oncology plays a more important role in recent years. Terms as “fit old,” “intermediate old,” and “frail old” were created to characterize elderly patients into categories determining their eligibility for therapy [8]. General assessment tools like ECOG or Karnofsky status and special tools for elderly patients like the Charlson Comorbidity Index (CCI) [9] were developed. However, none of these scores is specialized for elderly patients and no consensus for patient selection for CRT for advanced age patients exists.

This article compares acute toxicities, treatment compliance, tumor control, and survival between younger patients and groups of ≥65, ≥70, and ≥75 years of age and attempts to answer the question if age alone is a prognostic factor for toxicities and oncological results following definitive CRT for SCCHN.

## 2. Patients and Methods

### 2.1. Patients

We retrospectively analyzed 158 patients treated with definitive CRT for histologically proven SCCHN between June 2007 and January 2015 at our department following institutional review board approval. All patients underwent physical examination, computed tomography/magnetic resonance imaging (CT/MRI) of the neck, panendoscopy with biopsy and CT of the thorax and abdomen or chest X-ray, and sonography of the abdomen before the start of treatment. All cases were discussed in an interdisciplinary tumor board prior to treatment decision and all patients provided informed consent for CRT. In 135 of 158 cases, pretreatment biopsies were screened for HPV-status by immunohistochemical staining of the surrogate protein p16.

### 2.2. Treatment Protocols

Radiotherapy was delivered using either three-dimensional conformal radiotherapy (*n* = 38) or, since 2010, intensity-modulated radiotherapy (IMRT, *n* = 120). All patients received planning CTs, thermoplastic masks were used for immobilization, and 6 MV photon energies were used for treatment. The planning target volumes (PTV) included elective irradiation of the draining cervical nodes up to 50 Gy, dose escalation up to 58–60 Gy for involved lymph node levels/levels at high risk, and a boost to the primary tumor with a median total reference dose of 70.6 Gy. Concurrent chemotherapy (*n* = 158, 100%) was applied at weeks 1 and 5 (days 1–5) and was platin-based with a cumulative cisplatin dose of 180–200 mg/m^2^ and 6000 mg/m^2^ 5-fluorouracil (5-FU) as continuous i.v. infusion for most (>70%) patients. Patients with impaired renal function received either a combination of mitomycin c (10 mg/m^2^ at weeks 1 and 5) and 5-FU, or carboplatin (5 days x AUC 1 at weeks 1 and 5) and 5-FU. Acute toxicities during treatment were scored using the Common Terminology Criteria for Adverse Events (CTCAE version 3.0).

### 2.3. Follow-Up

The initial treatment response was evaluated 6–12 weeks after the completion of CRT via physical examination and CT/MRI of the head and neck region. Follow-up examinations, panendoscopy, and biopsy in case of suspicious findings and CT/MRI scans of the head and neck were scheduled every 3 months for the first 2 years and every 6 months thereafter for a total of 5 years.

### 2.4. Statistical Analysis

Differences between groups were assessed using Fisher's exact test or Pearson's chi-squared test for categorical variables. The main outcome measures were LRC, distant metastases-free survival (DMFS), progression-free survival (PFS), and OS. Cumulative incidences and survival times were calculated from the start of CRT/RT to the dates of respective events or last follow-up. Differences for cumulative incidences in terms of LRC, DMFS, PFS, and OS between the different age groups were assessed using the log-rank test. Uni- and multivariate Cox regression were carried out to assess the influence of several clinicopathological parameters on clinical outcomes. Factors significant in the univariate analysis were included for multivariate analysis and a forward selection method was used. Statistical analysis was carried out using SPSS 21. A *p* value < 0.05 was considered significant.

## 3. Results

### 3.1. Patient and Treatment Characteristics

A total of 158 patients (122 men, 77%) were treated with definitive CRT (*n* = 158) for histologically proven SCCHN between June 2007 and January 2015 at our department. Patients and tumor characteristics are summarized in Table 1. The median age was 61 (range, 36–91). Tumors were mostly located in the oropharynx and oral cavity (39% and 31%, resp.). A total of 100 patients (63%) were younger than 65 years. P16 status was assessed in 135 patients with no significant difference for the younger cohort (*p* = 0.201). History of smoking was assessed for 116 individuals with no difference between the groups (*p* = 0.533). The younger cohort contained significantly more men (*p* = 0.01), but did not differ significantly for tumor size, nodal staging, and grading from the elderly patients. Median RT dosage and the chosen regimen of concurrent chemotherapy did not differ between the groups: in both age cohorts, the percentage of patients that received platin-based chemotherapy was between 70% and 80%. The total dose of chemotherapy administered to the patients as percentage of the prescribed dosage and the patients ECOG status were also balanced between the groups (*p* values: 0.458 and 0.173, resp.).

### 3.2. Acute Hematological and Nonhematological Toxicities

The acute toxicities of the 158 patients according to CTCAE scoring, separated between age groups, are shown in Table 2. Leucocytopenia ≥ grade 3 was the only side effect differing significantly between the age groups, with older patients showing less events than younger patients (*p* values 0.043 and 0.017 for patients ≥ 65 and ≥70 versus younger, resp.). Anemia and thrombocytopenia did not differ between the groups. Regarding the nonhematological toxicities like dermatitis, dysphagia, mucositis, and pain, no significant differences were found.

### 3.3. Tumor Control and Survival

**Figure 1:**
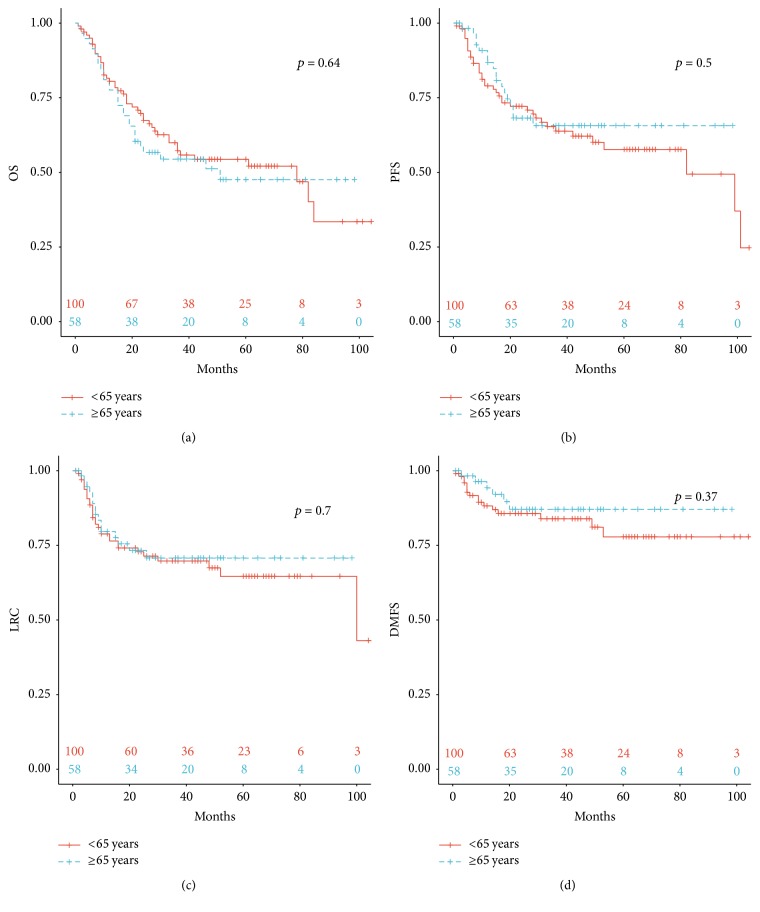
Overall survival (OS), progression-free survival, locoregional control (LRC), and distant metastasis-free survival (DMFS) according to age; log rank (Mantel Cox).

The clinical outcomes of the 158 patients are summarized in Figure 1 and Table 3. The Kaplan-Meier curves show OS, PFS, LCR, and DMFS stratified by age < 65 and ≥65 years (Figure 1). No significant differences were found for the above endpoints (*p* = 0.640; *p* = 0.500; *p* = 0.700; and *p* = 0.370, resp.) after a median follow-up of 29 months (range, 1–115). The table shows clinical outcomes after 3 years of follow-up. Also for the groups of ≥65 (*n* = 58), ≥70 (*n* = 33), and ≥75 (*n* = 16) versus the younger cohorts, no significant differences regarding the 3-year-outcomes were noted. When compared with the younger patients, *p* values for the group of ≥65 for OS, PFS, LCR, and DMFS were 0.682, 0.246, 0.530, and 0.282; for the group of ≥70 0.765, 0.746, 0.882, and 0.335; and for the group of ≥75 0.750, 0.782, 0.801, and 0.819, respectively.

### 3.4. Clinicopathological Factors Influencing Tumor Control and Survival

To identify clinicopathological factors influencing OS, PFS, LCR, and DMFS, univariate and multivariate analyses were carried out (Table 4). In univariate analysis, history of smoking (smoker versus never-smoker) was associated with higher cumulative incidence of death (smoker/nonsmoker, *p* = 0.032) and more distant failures (smoker/nonsmoker, *p* = 0.044). Smoking also remained significant in multivariate analysis regarding OS (*p* = 0.005) and DMFS (*p* = 0.029). More advanced N-stage disease was found to be a significant prognostic factor for OS (*p* = 0.024), PFS (*p* = 0.047), and DMFS (*p* = 0.047). In multivariate analysis advanced N-stage disease only remained significant for DMFS (*p* = 0.014). Worse ECOG performance score (ECOG2-3/ECOG0-1) was associated with poorer OS (*p* = 0.005), PFS (*p* = 0.033), and LCR (*p* = 0.011). The factor remained significant in multivariate analysis for OS (*p* = 0.004) and PFS (*p* = 0.048).

## 4. Discussion

Despite an increasing incidence of SCCHN in younger individuals [10], most patients present in a more advanced age [8]. As the median age at diagnosis currently amounts to 63 years [11], it is evident that in a continuously aging population the adequate treatment of these patients is a growing challenge for head and neck surgeons and radiation oncologists.

According to most definitions, individuals with ≥65 years of age are considered as “elderly” [12] and the National Institute of Aging also uses the subcategories “older old” and “oldest old” (for people between 75 and 85 and older than 85 years, resp.) [13]. In the present study, we analyzed our single-center experience in treating patients with definitive, concomitant CRT after dividing our entire cohort in various age-specific subgroups. As the patients treated with CRT older than 75 years of age (“older and oldest old”) were very few, we analyzed our results separately for patients with ≥65, ≥70, and ≥75 years of age. We did not observe any significant differences regarding survival and local and distant tumor control for any of the aforementioned groups. Severe treatment-related adverse events (grade ≥ 3) were also statistically not different between the age cohorts, with the exception of leukocytopenia which was intriguingly somewhat more common in younger patients. This finding could possibly be explained by bias of our retrospective analysis as chemotherapy may have been more often interrupted at the beginning of a decrease in the leucocyte numbers in elderly patients. Nevertheless, the percentages of patients that completed the prescribed chemotherapy were similar between the age groups (Table 1).

The data comparing outcome and treatment complications for various age groups in the current literature originate from either retrospective or non-preplanned analyses of prospective studies and are inconsistent and conflicting.

It is generally believed that the oncological results and tolerability of head and neck surgeries are similar for younger and older SCCHN patients, although some authors indicate slightly increased complication rates for the elderly [14, 15]. A recent, large retrospective analysis of more than 1400 patients with outpatient thyroidectomy revealed no differences compared to the control cohort, even for “super-elderly” patients [16]. Concerning the reconstructive surgery with free flaps, many modern publications show that the procedure is feasible for elderly patients with no substantial disadvantages and that performance status rather than age alone was predictive of complications [17–19]. Intriguingly, a recent patterns-of-care analysis showed that older patients were far more commonly treated with nonsurgical methods like radiotherapy and CRT [20], which makes careful considerations about feasibility and results of these modalities even more important.

With respect to RT and CRT, the data are more conflicting and recent cancer registry analyses provide contradictory results [21–23]. A meta-analysis by Pignon et al., 1996 [24] on 1589 patients with head and neck cancer enrolled in EORTC trials and treated with RT/CRT showed that OS and most toxicities were similar for younger and elderly patients when examined in different age ranges from 50 to 75 years and over. This analysis concludes that chronological age is not relevant for treatment decisions. These results are consistent with our observations and interestingly the objective acute mucositis rates also showed no significant difference, resembling our results. A further meta-analysis conducted by the same authors in 2009 [25] on 17.346 SCCHN patients treated between 1965 and 2000 with simultaneous CRT showed a decreasing effect of chemotherapy at higher ages. In contrast to that, in terms of larynx preservation the benefit of concomitant chemotherapy seems to be consistent for all age groups [4], although late toxicity was more common in the elderly [7]. In our present study, almost one-third of the patients also presented with locally advanced hypopharynx or larynx carcinoma and such were treated for larynx preservation, but a difference to the large larynx preservation trial above [6] was that we mostly used a chemotherapy regimen with a lower prescribed cisplatin dose, often in combination with 5FU. In the largest published database analysis about elderly SCCHN patients treated with radiotherapy [26], 4165 patients were analyzed. The authors could not identify any age threshold above which systemic treatment did not show a survival benefit and concluded that chemotherapy could and should be used for all age groups. However, contradictory to our data, the data-based analysis defined the elderly as older than 71 years, did not differentiate between concurrent and other forms of chemotherapy (e.g., solely induction), and also included patients without any systemic therapy after nodal surgery or even with nasopharyngeal carcinoma. The authors also did not report any toxicities making altogether the interpretation and comparison of the results with our study difficult.

Another retrospective, single-center analysis could not identify any differences in the rates of treatment interruption, completion, and treatment-related deaths for younger versus older patients treated with intensified nonsurgical procedures such as CRT and altered fractionation [27]. This observation is matching our results and underlines the feasibility of such therapeutical procedures. Nevertheless, in the latest study of Huang et al., the cause specific survival for the elderly cohort was satisfying but somewhat worse when compared to that of the younger cohort. Such differences may be attributed to the different biology of tumors in various age groups, as SCCHN in younger individuals is more commonly attributed to HPV infection, a status associated with improved tumor control and survival [28]. Furthermore, a recent investigation of the impact of HPV on the prognosis of elderly patients confirmed this relationship also for patients older than 70 years [29]. In our patient collective, the proportion of p16-positive patients was similar for all groups and no significant differences regarding the oncological endpoints could be found between p16-positive and p16-negative patients. The second observation appears somewhat surprising, but could be explained when the relatively small incidence of p16-positive patients (ca. 20%) and at the same time the high amount of smokers in the present study are taken into account: it has been already demonstrated that HPV-positive smokers have an impaired prognosis compared to HPV-positive nonsmokers [28]. Smoking status was also an independent prognosticator for worse OS and DMFS for the entire patient cohort, a correlation that has been demonstrated before [30, 31]. Caparrotti et al. could only show a trend for impaired outcome for elderly smokers [29]. The only pathological parameter with an impact on the endpoints was the nodal stage, one of the strongest known prognostic parameters in SCCHN with strong negative association with metastases-free survival [32–34], possibly as indicator of circulating tumor cells [35].

Interestingly, the only clinical factor with a significant impact on survival in the multivariate analysis was the ECOG performance status, indicating again that the general condition of the patient remains a far more reliable parameter that the chronological age. In elderly SCCHN patients, comorbidities are an independent predictor of survival [29, 36–38]. The Comprehensive Geriatric Assessment (CGA) [39] and the Geriatric-8 screening tool (G8) [40] also take functional, mental, and psychological status of the patient into account and could help differentiate patients suitable for intensified treatment. In any case, CRT for older SCCHN patients should be performed with maximum supportive care [41] and preferably in high-volume centers where modern approaches such as intensity-modulated radiotherapy could help improving outcomes [42].

Finally, novel agents such as targeted therapies and immunotherapy could help decreasing toxicities without compromising tumor control. In a trial by Bonner et al., Cetuximab, an epidermal-growth-factor receptor-inhibitor, enhanced the therapeutic effect of radiotherapy when applied simultaneously [43]. The effect was limited to younger patients (<65 years), but the trial was not powered to answer this question. Nevertheless, Cetuximab did not negatively affect the quality of life [44] and could be an option for elderly patients. In the future, a combination of radiotherapy and immunotherapy (e.g., with PD-1 inhibitors) could advance to a new standard for patients not amenable to CRT [45], as they do not show any negative impact on quality of life, at least in the palliative setting [46].

Altogether, there is enough experience showing that elderly SCCHN patients can withstand an intensive, curative treatment without experiencing a more pronounced decline in the quality of life than younger ones [47, 48] and the biggest danger relies on undertreating these patients [45].

The main strength of the present work is the homogenous collective analyzed, treated solely with concurrent chemoradiotherapy for a single histology at a single institution. This study has also some limitations: first of all the retrospective nature of the study makes it sensitive for bias, such as selection bias. For example, it is possible that some very frail patients, who were in advance excluded from a CRT, were more often older than 65 years. Secondly, important factors, such p16 and smoking, could be assessed for the majority, but not for all patients. However, we think that we could provide some useful results, underlying the fact that intensified treatment with CRT is feasible and effective, even in advanced age, if performance status and comorbidities are taken into account.

## Figures and Tables

**Table 1:** Patient characteristics; ^*∗*^Fisher exact test (2-sided); ^*∗∗*^Pearson's chi-squared test.

Patient characteristics			
Characteristics			*p* value
Total number	158		
Median age	61 (range 36–91)		

	*n (%)*		

Age ≥ 65	58 (37)		
Age ≥ 70	33 (21)		
Age ≥ 75	16 (10)		
Male	122 (77)		

	Age < 65 *n* (%)	Age ≥ 65, *n* (%)	

Number	100 (63)	58 (37)	
Tumor localization			
Oral cavity	31 (31)	18 (32)	0.543^*∗∗*^
Oropharynx	42 (42)	20 (34)	
Hypopharynx, larynx	27 (27)	20 (34)	

p16 positivity	19/82 (16)	14/53 (24)	0.686^*∗*^
(available for *n* = 135, 85%)			
History of smoking	50/75 (67)	30/41 (73)	0.533^*∗*^
(available for *n* = 116, 73%)			
Male	84 (84)	38 (66)	0.01^**∗**^
T2	5 (5)	4 (7)	0.794^*∗∗*^
T3	24 (24)	16 (28)	
T4	71 (71)	38 (66)	
N0	6 (6)	10 (17)	0.379^*∗∗*^
N1	3 (3)	0 (0)	
N2	78 (78)	45 (78)	
N3	4 (4)	1 (2)	
G1	7 (7)	2 (3)	0.069^*∗∗*^
G2	77 (77)	38 (66)	
G3	16 (16)	18 (31)	

RT dose (Gy)	70.6 (59–72.6)	70.6 (50–74.6)	0.59^*∗∗*^
Median (range)			
Concomitant chemotherapy (*n* = 158, 100%)			
Platin-based chemotherapy	72 (72)	43 (74)	0.854^*∗*^
Chemotherapy completion			
100%	64 (64)	32 (55)	0.458^*∗∗*^
≥50%	31 (31)	20 (35)	
<50%	5 (5)	6 (10)	
ECOG performance status			
0	34 (34)	13 (22)	0.173^*∗∗*^
1	48 (48)	27 (47)	
2	12 (12)	8 (14)	
3	1 (1)	2 (3)	
Not reported	6 (6)	8 (14)	

**Table 2:** Acute hematological and nonhematological toxicities according to CTCAE version 3.0; Fisher exact test (2-sided).

Acute CTC-grade 3+ toxicities									
	(%)			(%)			(%)		
	Age < 65	Age ≥ 65	*p* value	Age < 70	Age ≥ 70	*p* value	Age < 75	Age ≥ 75	*p* value
Leucocytopenia	26	12	***0.043***	25	6	***0.017***	22	6	0.196
Anaemia	9	3	0.331	8	3	0.461	8	0	0.605
Thrombocytopenia	9	3	0.331	8	3	0.461	7	6	1
Dermatitis	15	17	0.822	16	15	1	17	6	0.471
Dysphagia	52	62	0.247	54	64	0.331	55	63	0.607
Mucositis	47	48	1	47	49	1	48	44	0.798
Pain	21	21	1	22	18	0.811	22	13	0.526

**Table 3:** Clinical outcomes at 3 years of follow-up, stratified by age; log rank (Mantel Cox).

3-year follow-up									
	(%)			(%)			(%)		
	Age < 65	Age ≥ 65	*p* value	Age < 70	Age ≥ 70	*p* value	Age < 75	Age ≥ 75	*p* value
Number (%)	100 (63)	58 (37)		125 (79)	33 (21)		142 (90)	16 (10)	
OS	49	50	0.682	49	51	0.765	49	50	0.75
PFS	55	67	0.246	58	63	0.746	59	65	0.782
LCR	63	71	0.53	65	66	0.882	66	65	0.801
DMFS	78	87	0.282	79	89	0.335	81	85	0.819

**Table 4:** Univariate and multivariate analysis of clinicopathological parameters regarding overall survival (OS), progression-free survival (PFS), local control rate (LCR), and distant metastasis-free survival (DMFS); ^*∗*^status for 135/158 patients available; ^*∗∗*^status for 116/158 patients available.

Categories	Univariate analysis											
	OS			PFS			LCR			DMFS		
	HR	CI 95%	*p* value	HR	CI 95%	*p* value	HR	CI 95%	*p* value	HR	CI 95%	*p* value
p16 status; neg/pos^*∗*^	1.65	0.83–3.28	0.153	2.351	0.996–5.548	0.051	2.088	0.811–5.371	0.127	6.044	0.806–45.305	0.08
Smoker/nonsmoker^*∗∗*^	1.846	1.054–3.233	**0.032**	1.503	0.777–2.906	0.226	1.387	0.664–2.895	0.384	2.840	1.029–7.835	**0.044**
Sex male/female	1.008	0.577–1.763	0.977	1.271	0.691–2.339	0.44	1.004	0.496–2.035	0.99	1.364	0.532–3.496	0.518
T3-4/T1-2	1.125	0.453–2.792	0.8	1.192	0.430–3.303	0.736	1.068	0.331–3.453	0.912	2.474	0.730–8.369	0.146
N3/N2/N0-1	2.004	1.098–3.659	**0.024**	2.002	1.008–3.974	**0.047**	1.498	0.732–3.062	0.268	3.104	1.016–9.483	**0.047**
G3/G2/G1	0.75	0.480–1.172	0.206	0,869	0.511–1.478	0.604	0,804	0.445–1.454	0.471	1.518	0.680–3.387	0.309
ECOG2-3/ECOG0-1	2.303	1258–4.129	**0.005**	2.092	1.060–4.129	**0.033**	2.589	1.246–5.380	**0.011**	1.550	0.521–4.615	0.431
Age < 65/≥65	0.895	0.558–1.436	0.646	1.310	0.737–2.327	0.357	1.131	0.606–2.110	0.699	0.651	0.255–1.666	0.371
Age < 70/≥70	0.912	0.524–1.589	0.746	1.057	0.545–2.049	0.871	0.892	0.441–1.806	0.751	0.59	0.174–1.994	0.396
Age < 75/≥75	0.87	0.417–1.814	0.71	1.069	0.426–2.684	0.887	0.833	0.328–2.115	0.701	0.918	0.214–3.932	0.909

Categories	Multivariate analysis											
	OS			PFS			LCR			DMFS		
	HR	CI 95%	*p* value	HR	CI 95%	*p* value	HR	CI 95%	*p* value	HR	CI 95%	*p* value

Smoker/nonsmoker^*∗∗*^	2.321	1.287–4.183	**0.005**							3.222	1.130–9.189	**0.029**
N3/N2/N0-1	2.356	0.947–5.863	0.066	1.819	0.859–3.852	0.118				6.890	1.479–32.109	**0.014**
ECOG2-3/ECOG0-1	2.573	1.344–4.928	**0.004**	1.990	1.007–3.933	**0.048**						
